# Risk of functional disability associated with solid fuel use and population impact of reducing indoor air pollution in China: A national cohort study

**DOI:** 10.3389/fpubh.2022.976614

**Published:** 2022-10-03

**Authors:** Ziyang Ren, Weidi Sun, Shiyi Shan, Leying Hou, Siyu Zhu, Qian Yi, You Wu, Chao Guo, Jufen Liu, Peige Song

**Affiliations:** ^1^School of Public Health and Women's Hospital, Zhejiang University School of Medicine, Hangzhou, China; ^2^Institute of Reproductive and Child Health/Key Laboratory of Reproductive Health, National Health Commission of the People's Republic of China, Peking University, Beijing, China; ^3^Department of Epidemiology and Biostatistics, School of Public Health, Peking University, Beijing, China; ^4^Department of Epidemiology, School of Public Health, Sun Yat-sen University, Guangzhou, China; ^5^School of Medicine, Tsinghua University, Beijing, China; ^6^Institute for Hospital Management, Tsinghua University, Shenzhen, China; ^7^Institute of Population Research, Peking University, Beijing, China; ^8^APEC Health Science Academy (HeSAY), Peking University, Beijing, China

**Keywords:** household fuel, solid fuel, indoor air pollution, functional disability, activity of daily living, instrumental activity of daily living

## Abstract

**Background:**

In China, numerous people still rely on solid fuel for household use. To date, the association between household solid fuel use and functional disability, and what benefit reducing household solid fuel usage could bring at the population level to China remain unclear.

**Method:**

Data were from the China Health and Retirement Longitudinal Study. Household fuel was classified as clean or solid for cooking or heating. Functional disability was defined as difficulties in any item of activities of daily living (ADL) or instrumental activities of daily living (IADL). The associations of household fuel use in 2011 and its transitions between 2011 and 2013 with subsequent ADL or IADL disability were assessed with Cox proportional-hazards models. The number of events prevented in a population (NEPP) was generated to estimate how many functionally disabled patients could be prevented by reducing solid fuel usage.

**Results:**

A total of 6,216 and 9,716 participants without prior ADL or IADL disability in 2011 were included. Solid (vs. clean) fuel users were more likely to develop ADL and IADL disability, with hazard ratios (HRs) and 95% confidence intervals (CIs) of 1.37 (1.28~1.45) and 1.38 (1.31~1.46) for using both solid cooking and heating fuel. Furthermore, participants that switched heating fuel from solid to clean (vs. keep solid) were about 20% less likely to develop functional disability. Cooking fuel use switching from solid to clean (vs. keep solid) was also negatively associated with IADL disability (HR = 0.84, 95% CI 0.74~0.96). Over the next 7 years, raising clean fuel usage to 80% could prevent about 4.9 million ADL disability and 2.6 million IADL disability among Chinese aged 45 and older.

**Conclusion:**

Household solid fuel use was a risk factor for functional disability. Reducing solid fuel usage could help reduce the burden of functional disability in the current aging society of China.

## Introduction

Population aging is now a significant global public health concern (1). Functional disability (FD), defined as limitations in activities of daily living (ADL) or instrumental activities of daily living (IADL), is significantly associated with aging and has reached a staggering proportion these years, with around 110 million individuals experiencing significant difficulties in functioning according to the World Health Survey (2–5). In recent years, it has also been discovered that air pollution can speed up aging and lead to FD (6, 7).

Indoor air pollution from the burning of household solid fuel for cooking and heating has received increasing attention recently (8–10). Although the Sustainable Development Goals (SDGs) seven issued by the United Nations calls for universal access to clean energy (11), there are still around 450 million people heavily relying on solid fuel for household requirements in China, especially the elderly and rural residents (12–14). Given the elderly typically spend the majority of their time indoors, their health is more likely to be impaired by prolonged exposure to indoor air pollution (15, 16).

Previous studies have found significant associations of solid fuel use with cognitive decline, visual impairment, and depression, all of which are risk factors for FD (17–19). Once functionally disabled, the elderly may spend more time indoors and be exposed to air pollution for longer, which may in turn aggravate their FD. Two cross-sectional studies have demonstrated associations of solid cooking fuel use with FD (20, 21). Wang et al. (22) also conducted a longitudinal study and found that using solid heating fuel was a risk factor for FD. However, limited studies have comprehensively investigated the longitudinal associations of household solid fuel use and its transition to cleaner with the occurrence and aggravation of FD.

To address this research gap, we hypothesize that long-term solid household fuel use is a risk factor for new-onset FD and that switching from solid to clean fuel can help prevent FD in middle-aged and elderly Chinese and conducted this study. Furthermore, we estimated how many potential FD cases could be prevented by abating indoor air pollution from household solid fuel.

## Method

### Study population

This study used data from the 2011–2018 China Health and Retirement Longitudinal Study (CHARLS), a nationally representative survey of adults aged 45 years and older from 450 villages/urban communities across China using a multistage probability sampling method (23). The national baseline survey of the CHARLS was conducted in 2011, with follow-ups in 2013, 2015, and 2018. Information on sociodemographic characteristics and health status were collected in each wave. Ethical approval was granted by the Institutional Review Board at Peking University. Each respondent has signed the written informed consent.

**Figure 1:**
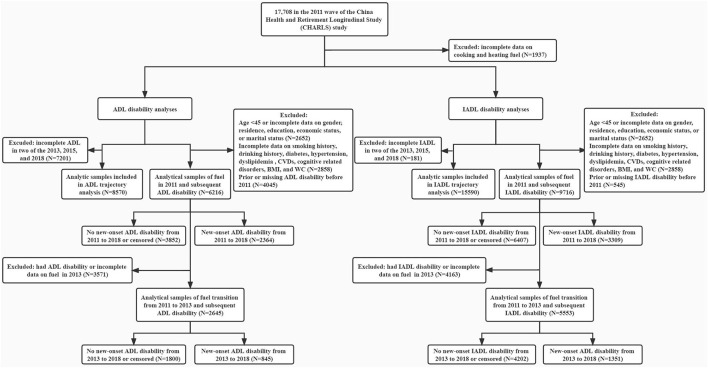
Flow chart. ADL, activity of daily living; IADL, instrumental activity of daily living; CVD, cardiovascular disease; BMI, body mass index; WC, waist circumference.

We enrolled participants with complete information on household cooking and heating fuel use, ADL/IADL at baseline, and at least two observations of ADL/IADL in the 2013, 2015, and 2018 waves for trajectory analyses. To investigate the long-term association between household fuel use in 2011 and new-onset ADL/IADL disability between 2011 and 2018, participants aged 45 years or older, with complete data on cooking and heating fuel use and covariates, and free of ADL/IADL disability at baseline were enrolled. To investigate the association of fuel use transition from 2011 to 2013 with subsequent new-onset ADL/IADL disability, we further excluded participants with incomplete information on cooking and heating fuel in 2013 or who had ADL/IADL disability before 2013 (Figure 1).

### FD assessment

In CHARLS, FD was assessed by ADL and IADL, which was derived from the participants' self-reported difficulty in the six ADL items (dressing, bathing, eating, getting into/out of bed, toileting, and controlling urination and defecation) and the five IADL items (meal preparation, shopping, doing housework, taking medicines and managing money) (2, 3). Each item had four answers, including “have no difficulty,” “have difficulty but can still do,” “have difficulty and need help,” and “cannot do.” Participants who reported difficulty in any of the six ADL items (or five IADL items) were defined as ADL/IADL disability. Furthermore, ADL/IADL disability were scored at each wave corresponding to the number of ADL/IADL items participants reported difficulty in. Accordingly, the sum of scores ranges from 0 to 6 and 0 to 5 for ADL/IADL disability.

### Household fuel usage

The CHARLS asked participants “What is the main source of cooking fuel/ heating energy?” Cooking and heating fuel was classified into clean fuel (i.e., solar, natural gas, marsh gas, liquefied petroleum gas, or electric) and solid fuel (i.e., coal, crop residue, and wood) in 2011 and 2013 (12, 19). The cooking and heating fuel use was denoted as “Both clean,” “Cooking clean and heating solid,” “Cooking solid and heating clean,” and “Both solid.” We further named the cooking and heating fuel use as “Both clean,” “1 solid,” and “Both solid” in analysis on household fuel use transition from 2011 to 2013.

### Covariates

Information on age, sex (male, female), residence (urban, rural), education (less than primary school, primary school, middle school, high school or above), marital status (married or cohabiting, single; “single” included participants who were separated from spouses, divorced, widowed, or unmarried), smoking history (never smoking, ever smoking), drinking history (never drinking, ever drinking), and cognitive-related disorders (no, yes) was collected through face-to-face interviews at baseline. Household economic status was assessed by the natural logarithm of per capita expenditures [ln (PCE)] and was categorized as bottom, middle, and top tertilec (24, 25).

Physical measurements, medical history, and blood biomarkers were recorded at baseline. Body mass index (BMI) status was divided into normal weight (BMI < 24.0 kg/m^2^), overweight (24 kg/m^2^ ≤ BMI < 28.0 kg/m^2^), and obesity (BMI ≥ 28.0 kg/m^2^). Waist circumference (WC) was defined as continuous. Hypertension was defined as blood pressure≥ 140/90 mmHg and/or self-reported diagnosis or treatment (26). Diabetes was defined as fasting plasma glucose ≥7.0 mmol/L, and/or random plasma glucose ≥11.1 mmol/L, and/or HbA1c ≥ 6.5%, and/or self-reported diagnosis or treatment (27). Dyslipidemia was defined as self-reported diagnosis or treatment, and/or total cholesterol (TC) ≥ 240 mg/dL, and/or low-density lipoprotein cholesterol (LDL-C) ≥ 160 mg/dL, and/or high-density lipoprotein cholesterol (HDL-C) ≤ 40 mg/dL, and/or triglycerides (TG) ≥ 200 mg/dL (28). Cardiovascular diseases (CVDs) and cognitive-related diseases were defined by self-reported diagnoses.

## Statistical analysis

The developmental trajectories of ADL/IADL disability scores from 2011 to 2018 were conducted by group-based trajectory modeling (GBTM), which can identify distinct groups of individuals with similar trajectories of a specific feature (29–31). Models were estimated with two to five groups and the best fitting model was selected with the lowest values of Akaike Information Criterion (AIC) and Bayesian Information Criterion (BIC) (Supplementary Table 1). The Chi-square test was further adopted to compare the household fuel among different trajectory groups.

The baseline characteristics of included participants were described as medians and interquartile ranges (IQRs) for continuous variables, and frequency and percent (%) for categorical variables. Wilcoxon rank sum tests for continuous variables and Chi-square tests for categorical variables were utilized to calculate *P*-values.

The Cox proportional-hazards model was used to investigate the association [hazard ratio (HR) and 95% confidence interval (CI)] of the cooking and heating fuel use in 2011 with new-onset ADL/IADL disability between 2011 and 2018 after adjusting for age, sex, residence, education, economic status, marital status, smoking history, drinking history, BMI status, WC, diabetes, hypertension, dyslipidemia, CVDs, and cognitive-related diseases in participants without ADL/IADL disability in 2011. The time to event was calculated as the intervals from the baseline survey dates (2011 to 2012) to the dates of the interview reporting an incident FD, death, loss of follow-up, or the end of follow-up (2013, 2015, or 2018), whichever came first. Mixed cooking and heating fuel use was further estimated using the floating absolute risk, which allows comparisons between any two exposure groups and can decrease undesired correlation between coefficients (32–34). Given the high usage of solid household fuel in rural areas, sex differences in exposure to solid fuel, and the pronounced hazards in the elderly, age- (<65 and ≥65), sex- (male and female), and residence-stratified (urban and rural) Cox proportional-hazards models were also conducted. To ensure the robustness of our results, sensitivity analysis was further conducted in individuals with no main chronic diseases. Considering that the time scales we used were not exact to a specific date, we also conducted a sensitivity analysis using logistic regression to verify the robustness of our conclusion. Furthermore, fully adjusted Cox proportional-hazard models and the floating absolute risk were also used to investigate the association of transition of household fuel use from 2011 to 2013 with subsequent new-onset ADL/IADL disability in participants without ADL/IADL disability in 2013, with time to event from the 2013 survey dates to the dates of the interview reporting an incident FD, death, loss of follow-up, or the end of follow-up (2015 or 2018), whichever came first.

Finally, we used the number of events prevented in a population (NEPP) to estimate the number of ADL/IADL disability cases that could be prevented over the next 7 years among the population aged 45 and above by reducing solid fuel. The NEPP describes the impact of interventions and can be used to estimate the incremental impact of moving from current to best practice (35, 36). The calculation formula is *NEPP* = *n* × *Id* × *Pe* × *r*_*u*_ × *HRR*, where *n* = population size of aged 45 years and above, Id = incidence density of FD from 2011 to 2018, Pe = the proportion eligible for intervention, r_u_ = the risk of FD in the group using solid fuel, and HRR = the hazard ratio reduction associated with the intervention, which was calculated as (*r*_*u*_ − 1)/*r*_*u*_. In order to reflect the incremental effect of changing from current to 'best' practice and to adjust for levels of compliance in the proportion eligible for intervention, Pe was calculated as (*Pb* − *Pt*) × *Pc*, where Pt = the proportion currently intervened, Pb = the proportion that would be intervened if best practice was adopted, and Pc = the proportion of the population who are adherent to their intervention. The data on population size of those aged 45 years and above (n) in 2020 were obtained from China Statistical Yearbook 2021 (37). Best practice intervention goals (Pb) were taken as 80% for clean fuel usage according to China's clean energy policy (38), and compliance with each intervention of clean fuel usage (Pc) was assumed to be 50%. Other indicators originated from our own study.

Reporting of this study was done in accordance with Strengthening the Reporting of Observational studies in Epidemiology (STROBE) guidelines. Analyses were performed using R statistical software version 4.1.2 (R Project for Statistical Computing) and STATA statistical software (version 15.0, STATA Corp). All analyses were two-sided, and a *P-*value of <0.05 or a 95% CI that did not cross 1.00 was considered statistically significant.

## Results

Eight thousand five hundred and seventy and 15,590 participants were included to identify the developmental trajectories of ADL/IADL disability scores between 2011 and 2018. The two distinct trajectories that best characterized the developmental courses of FD scores were finally selected and labeled as “Maintained low ADL/IADL score” and “Increasing ADL/IADL score” (Supplementary Figures 1, 2). Supplementary Tables 2, 3 show that solid fuel (vs. Clean) users were more likely to develop FD (all *P* Value < 0.001).

A total of 6,216 and 9,716 participants without prior ADL/IADL disability before 2011 were included, of whom 2,364 (38.0%) and 3,309 (34.1%) developed new-onset ADL/IADL disability during 2011 and 2018. The geographic distributions of the included participants are shown in Supplementary Figures 3, 4. The baseline characteristics of the included participants categorized by new-onset FD status are described in Supplementary Tables 4, 5.

The associations of household fuel used in 2011 with new-onset FD between 2011 and 2018 are shown in Tables 1, 2. Solid (vs. clean) cooking fuel users were more likely to develop incident ADL/IADL disability, with fully adjusted HRs (95% CIs) of 1.14 (1.04~1.26) and 1.27 (1.17~1.38). Participants who used solid heating fuel (vs. clean) were also 1.28 and 1.21 times more likely to develop new-onset ADL and IADL, respectively. For those using clean cooking fuel but solid heating fuel, the risks of ADL/IADL disability were elevated by 25% (HR = 1.25, 95% CI 1.14~1.36) and 8% (HR = 1.08, 95% CI 1.00~1.16). Furthermore, individuals who used both solid fuel (vs. both clean) demonstrated approximate 1.4 times higher risks of incident FD. In the age-, sex-, residence-stratified analyses, we found that the associations of fuel use with ADL were not modified by age and sex, while a stronger association between using solid heating fuel and ADL was observed among those who lived in urban area than that in rural area. We also found the associations of fuel use with IADL were not modified by residence, while stronger associations of using solid cooking fuel with IADL were observed among those who <65 than ≥65 and of using solid heating fuel with IADL among males than females. In the sensitivity analysis where we restricted to those without previous main chronic diseases (Supplementary Table 6), the associations were generally attenuated and turn to be non-statistically significant compared to the results from primary analysis. In Supplementary Table 7, the logistic regression of fuel usage in 2011 with new-onset ADL and IADL disability from 2011 to 2018 showed similar results in line with our primary analysis.

**Table 1:** HR (95% CI) of fuel usage with new-onset ADL disability: Cox proportional-hazards model.

	**Total** **population** **(*N* = 6,216)**	**Age-stratified**			**Sex-stratified**			**Residence-stratified**		
		**Age < 65** **(*N* = 4,417)**	**Age ≥ 65** **(*N* = 1,799)**	**P for interaction**	**Male** **(*N* = 2,714)**	**Female** **(*N* = 3,502)**	**P for interaction**	**Rural** **(*N* = 4,316)**	**Urban** **(*N* = 1,900)**	**P for interaction**
**Cooking fuel**				0.806			0.365			0.126
Clean	Reference	Reference	Reference		Reference	Reference		Reference	Reference	
Solid	1.14 (1.04~1.26)	1.12 (1.00~1.25)	1.16 (0.98~1.37)		1.20 (1.03~1.40)	1.10 (0.98~1.25)		1.09 (0.97~1.22)	1.24 (1.06~1.45)	
**Heating fuel**				0.378			0.061			0.048
Clean	Reference	Reference	Reference		Reference	Reference		Reference	Reference	
Solid	1.28 (1.14~1.45)	1.26 (1.09~1.46)	1.26 (1.03~1.56)		1.50 (1.23~1.84)	1.17 (1.01~1.36)		1.16 (0.99~1.37)	1.42 (1.19~1.70)	
**Cooking and heating fuel**				0.749			0.055			0.158
Both clean	1.00 (0.88~1.14)	1.00 (0.86~1.17)	1.00 (0.80~1.25)		1.00 (0.81~1.23)	1.00 (0.85~1.18)		1.00 (0.83~1.21)	1.00 (0.85~1.18)	
Cooking clean and heating solid	1.25 (1.14~1.36)	1.27 (1.14~1.41)	1.15 (0.98~1.34)		1.29 (1.12~1.49)	1.22 (1.10~1.37)		1.07 (0.95~1.20)	1.36 (1.19~1.55)	
Cooking solid and heating clean	1.04 (0.83~1.29)	1.08 (0.82~1.41)	0.92 (0.64~1.33)		0.68 (0.43~1.07)	1.22 (0.95~1.57)		0.91 (0.70~1.18)	1.01 (0.68~1.51)	
Both solid	1.37 (1.28~1.45)	1.33 (1.24~1.44)	1.33 (1.20~1.49)		1.50 (1.37~1.65)	1.29 (1.19~1.39)		1.16 (1.10~1.23)	1.57 (1.39~1.77)	

ADL, activities of daily living; HR, hazard ratio; CI, confidence interval; HR was adjusted for age, sex (male or female), residence (urban or rural), education (less than primary school, primary school, middle school, or high school or above), economic status (poor, middle, or rich), marital status (married or cohabiting, or single), smoking history (never smoking or ever smoking), drinking history (never drinking or ever drinking), body mass index status (normal weight, overweight, or obesity), waist circumference, diabetes (yes or no), hypertension (yes or no), dyslipidemia (yes or no), cardiovascular diseases (yes or no), and cognitive-related disorders (yes or no).

**Table 2:** HR (95% CI) of fuel usage with new-onset IADL disability: Cox proportional-hazards model.

	**Total population (*N* = 9716)**	**Age-stratified**			**Sex-stratified**			**Residence-stratified**		
		**Age < 65** **(*N* = 7495)**	**Age ≥ 65** **(*N* = 2221)**	**P for interaction**	**Male** **(*N* = 5043)**	**Female** **(*N* = 4673)**	**P for interaction**	**Rural** **(*N* = 6441)**	**Urban** **(*N* = 3275)**	**P for interaction**
**Cooking fuel**				0.021			0.338			0.079
Clean	Reference	Reference	Reference		Reference	Reference		Reference	Reference	
Solid	1.27 (1.17~1.38)	1.32 (1.20~1.45)	1.15 (0.99~1.34)		1.30 (1.15~1.46)	1.25 (1.13~1.39)		1.22 (1.10~1.34)	1.37 (1.19~1.56)	
**Heating fuel**				0.394			0.005			0.827
Clean	Reference	Reference	Reference		Reference	Reference		Reference	Reference	
Solid	1.21 (1.10~1.33)	1.23 (1.10~1.39)	1.17 (0.98~1.40)		1.41 (1.21~1.65)	1.08 (0.95~1.23)		1.21 (1.06~1.38)	1.20 (1.03~1.39)	
**Cooking and heating fuel**				0.154			0.005			0.337
Both clean	1.00 (0.90~1.11)	1.00 (0.88~1.13)	1.00 (0.82~1.22)		1.00 (0.85~1.18)	1.00 (0.87~1.15)		1.00 (0.85~1.17)	1.00 (0.87~1.15)	
Cooking clean and heating solid	1.08 (1.00~1.16)	1.08 (0.99~1.18)	1.09 (0.95~1.25)		1.17 (1.04~1.31)	1.01 (0.92~1.12)		1.10 (1.00~1.22)	1.03 (0.91~1.16)	
Cooking solid and heating clean	1.15 (0.98~1.35)	1.18 (0.97~1.44)	1.05 (0.79~1.39)		0.89 (0.67~1.18)	1.33 (1.09~1.62)		1.13 (0.93~1.37)	1.16 (0.87~1.55)	
Both solid	1.38 (1.31~1.46)	1.43 (1.35~1.52)	1.26 (1.14~1.39)		1.52 (1.40~1.64)	1.28 (1.20~1.37)		1.34 (1.27~1.41)	1.46 (1.31~1.62)	

ADL, activities of daily living; IADL, instrumental activities of daily living; HR, hazard ratio; CI, confidence interval. HR was adjusted for age, sex (male or female), residence (urban or rural), education (less than primary school, primary school, middle school, or high school or above), economic status (poor, middle, or rich), marital status (married or cohabiting, or single), smoking history (never smoking or ever smoking), drinking history (never drinking or ever drinking), body mass index status (normal weight, overweight, or obesity), waist circumference, diabetes (yes or no), hypertension (yes or no), dyslipidemia (yes or no), cardiovascular diseases (yes or no), and cognitive-related disorders (yes or no).

**Figure 2:**
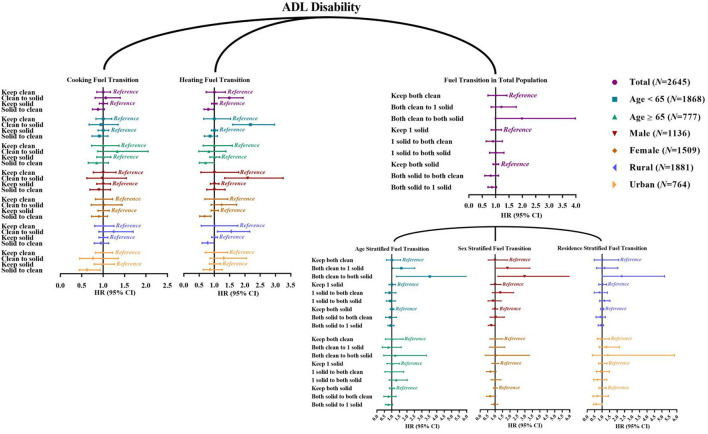
Hazard ratio (95% confidence interval) of fuel usage transitions with new-onset ADL disability: Cox proportional-hazards model. ADL, activities of daily living. The reference of “Clean to solid” is “Keep clean;” the reference of “Solid to clean” is “Keep solid;” the reference of “Both clean to 1 solid” and “Both clean to both solid” is “Keep both clean;” the reference of “1 solid to both clean” and “1 solid to both solid” is “Keep 1 solid;” the reference of “Both solid to both clean” and “Both solid to 1 solid” is “Keep both solid”.

**Figure 3:**
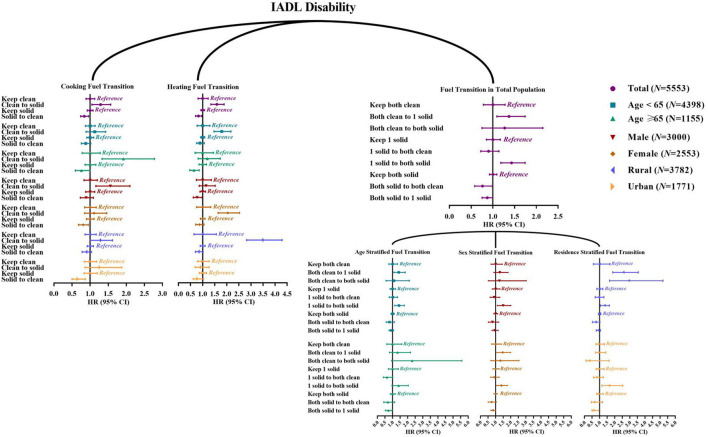
Hazard ratio (95% confidence interval) of fuel usage transitions with new-onset IADL disability: Cox proportional-hazards model. IADL, instrumental activities of daily living. The reference of “Clean to solid” is “Keep clean;” the reference of “Solid to clean” is “Keep solid;” the reference of “Both clean to 1 solid” and “Both clean to both solid” is “Keep both clean;” the reference of “1 solid to both clean” and “1 solid to both solid” is “Keep 1 solid;” the reference of “Both solid to both clean” and “Both solid to 1 solid” is “Keep both solid”.

Supplementary Figures 5, 6 presented the household fuel use transition from 2011 to 2013 in participants without ADL/IADL disability before 2013 and their follow-up status between 2013 and 2018. Baseline characteristics of these participants were also described in Supplementary Tables 8, 9. We found that heating fuel use switching from clean to solid (vs. keep clean) was significantly associated with incident ADL disability (HR = 1.49, 95% CI 1.14~1.94). In contrast, individuals with the transition from heating solid to clean (vs. keep solid) were less likely to develop ADL disability (HR = 0.80, 95% CI 0.66~0.98). For cooking fuel usage, significant association was found of transition from solid to clean (vs. keep solid) with a decreased risk of new-onset ADL disability among urban residents (Figure 2; Supplementary Table 10). We also observed a significant association of household fuel usage switching from both clean to 1 solid and to both solid (vs. keep both clean) with new-onset ADL disability among those aged <65 years, with HRs (95%CIs) of 1.63 (1.05~2.53) and 3.53 (1.31~9.53). In terms of IADL, cooking fuel use switching from clean to solid (vs. keep clean), heating fuel usage switching from clean to solid (vs. keep clean), transition from both clean to 1 solid (vs. keep both clean), and from 1 solid to both solid (vs. keep 1 solid) were positively associated with incident IADL disability, while cooking fuel usage switching from solid to clean (vs. keep solid), heating fuel usage switching from solid to clean (vs. keep solid), transition from both solid to 1 solid (vs. keep both solid), and from both solid to both clean (vs. keep both solid) were negatively associated with incident IADL disability (Figure 3; Supplementary Table 11).

Finally, the NEPP for incident FD are shown in Table 3. By increasing clean cooking fuel usage up to 80%, about 0.9 million aged <65, 0.9 million aged ≥65, 1.3 million males, 0.6 million females, 0.4 million urban residents, 1.4 million rural residents, and a total of 1.8 million people aged 45 and older could be prevented from ADL disability over the next 7 years. In terms of heating fuel usage, up to 4.9 million cases of ADL disability would be prevented. Moreover, 2.2 million and 2.6 million patients with IADL disability could be prevented under the same goal for cooking and heating fuel usage. Even if the compliance with the intervention was reduced to 50%, the number of individuals able to be prevented could reach half of the ideal goal.

**Table 3:** Number of cases of ADL or IADL that could be prevented when prevalence levels of “clean fuel usage for up to 80%” in a Chinese population.

**Outcome**	**Stratified** **population**	**N /10^5^**	**Id /10^5^**	**Pb**	**Pt**	**Pc**	**Pe**	**ru**	**HRR**	**NEPP**
ADL	**Cooking fuel**									
	**Age-stratified**									
	<65	4101.72907	8473.824	0.80	0.361	0.50	0.219	1.12	0.107	914765.837
	≥65	1906.3528	11634.349	0.80	0.313	0.50	0.243	1.16	0.138	863817.863
	ALL	6008.082	9377.475	0.80	0.347	0.50	0.226	1.14	0.123	1784928.598
	**Sex-stratified**									
	Male	2974.94668	9000.188	0.80	0.328	0.50	0.236	1.20	0.167	1264942.695
	Female	3033.13519	9651.401	0.80	0.363	0.50	0.219	1.10	0.091	639956.940
	All	6008.082	9377.475	0.80	0.347	0.50	0.226	1.14	0.123	1784928.598
	**Residence-stratified**									
	Rural	2517.21544	22013.898	0.80	0.242	0.50	0.279	1.09	0.083	1390380.709
	Urban	3490.8664	3909.220	0.80	0.586	0.50	0.107	1.24	0.194	350579.734
	All	6008.082	9377.475	0.80	0.347	0.50	0.226	1.14	0.123	1784928.589
	**Heating fuel**									
	**Age-stratified**									
	<65	4101.72907	8473.824	0.80	0.177	0.50	0.311	1.26	0.206	2813183.793
	≥65	1906.3528	11634.349	0.80	0.170	0.50	0.315	1.26	0.206	1816746.498
	All	6008.082	9377.475	0.80	0.175	0.50	0.312	1.28	0.219	4927968.813
	**Sex-stratified**									
	Male	2974.94668	9000.188	0.80	0.164	0.50	0.318	1.50	0.333	4258686.396
	Female	3033.13519	9651.401	0.80	0.184	0.50	0.308	1.17	0.145	1532542.950
	All	6008.082	9377.475	0.80	0.175	0.50	0.312	1.28	0.219	4927968.813
	**Residence-stratified**									
	Rural	2517.21544	22013.898	0.80	0.114	0.50	0.343	1.16	0.138	3039897.168
	Urban	3490.8664	3909.220	0.80	0.314	0.50	0.243	1.42	0.296	1393584.560
	All	6008.082	9377.475	0.80	0.175	0.50	0.312	1.28	0.219	4927968.789
IADL	**Cooking fuel**									
	**Age-stratified**									
	<65	4101.72907	6015.909	0.80	0.421	0.50	0.189	1.32	0.242	1495817.808
	≥65	1906.3528	10035.143	0.80	0.330	0.50	0.235	1.15	0.130	674944.430
	All	6008.082	6869.944	0.80	0.400	0.50	0.200	1.27	0.213	2227714.687
	**Sex-stratified**									
	Male	2974.94668	5687.722	0.80	0.398	0.50	0.201	1.30	0.231	1021574.153
	Female	3033.13519	8208.394	0.80	0.403	0.50	0.198	1.25	0.200	1235151.699
	All	6008.082	6869.944	0.80	0.400	0.50	0.200	1.27	0.213	2227714.687
	**Residence-stratified**									
	Rural	2517.21544	7481.164	0.80	0.286	0.50	0.257	1.22	0.180	1065519.839
	Urban	3490.8664	5664.797	0.80	0.625	0.50	0.087	1.37	0.270	638684.062
	All	6008.082	6869.944	0.80	0.400	0.50	0.200	1.27	0.213	2227714.676
	**Heating fuel**									
	**Age-stratified**									
	<65	4101.72907	6015.909	0.80	0.214	0.50	0.293	1.23	0.187	1662241.873
	≥65	1906.3528	10035.143	0.80	0.184	0.50	0.308	1.17	0.145	1001832.064
	All	6008.082	6869.944	0.80	0.207	0.50	0.296	1.21	0.174	2568712.203
	**Sex-stratified**									
	Male	2974.94668	5687.722	0.80	0.202	0.50	0.299	1.43	0.301	2173976.019
	Female	3033.13519	8208.394	0.80	0.213	0.50	0.294	1.12	0.107	877533.463
	All	6008.082	6869.944	0.80	0.207	0.50	0.296	1.21	0.174	2568712.203
	**Residence-stratified**									
	Rural	2517.21544	7481.164	0.80	0.136	0.50	0.332	1.20	0.167	1251319.736
	Urban	3490.8664	5664.797	0.80	0.348	0.50	0.226	1.21	0.174	937756.818
	All	6008.082	6869.944	0.80	0.207	0.50	0.296	1.21	0.174	2568712.190

N, population size of Chinese aged 45 years and above in 2020; Id, incidence density of functional disability in the population from 2011 to 2018; Pb, proportion be intervened when best practice adopted; Pt, proportion of those with the disability currently receiving intervention; Pc, compliance with intervention; Pe, proportion eligible for intervention; ru, risk of functional disability in the solid fuel usage group; HRR, hazard ratio reduction; NEPP, number of events prevented in the population.

## Discussion

In this longitudinal population-based study, we found that exposure to solid cooking fuel, solid heating fuel, and both of them were positively associated with incident FD. We also found that cooking and heating fuel use switching from clean to solid was a risk factor for incident FD while switching from solid to clean was associated with decreased risks of FD. Additionally, our results showed that reducing solid cooking fuel usage could prevent about 1.8 million and 2.2 million patients aged 45 and older with ADL/IADL disability in the 7-year follow-up. As for reducing solid heating fuel usage, 4.9 million ADL disability and 2.6 million IADL disability could be prevented.

Our findings are in accordance with and extend results from previous studies of household solid fuel use in association with FD. Though several studies have investigated the association between solid fuel use and FD (20–22), their findings were limited by the cross-sectional study design or the incomplete definition of household fuel.

In households with little access to clean fuels, solid fuels are usually burned in inefficient combustion devices like traditional stoves, in which solid fuels are hard to fully burn (39). The incomplete combustion emits kinds of hazardous pollutants such as nitrogen dioxide, carbon monoxide, and volatile organic compounds (40, 41). Long-term exposure to these toxic pollutants, however, may increase inflammatory cytokines in systemic circulation and affect the central nervous system, leading to cognitive decline and depression (42, 43). These nervous impairments will subsequently cause a loss of hand-grip strength (44) and accelerate frailty (45). All of these disorders, if not treated well, can finally lead to FD, especially in older adults (46–48).

Noteworthy, switching from solid to clean household fuel was negatively associated with new-onset FD, implying that reducing solid household fuel use may effectively prevent FD. In March 2022, the Chinese government introduced a 5-year plan on elderly care, emphasizing the importance of preventing FD in older adults (49). This study provides population-based evidence for policymakers and highlights the benefits of reducing household solid fuel for FD prevention. In other words, we urge the government to elevate the usage of clean fuel to 80% in houses to prevent potential FD.

To the best of our knowledge, we are the first to investigate the longitudinal associations of household fuel use and its transition with new-onset FD in China. We employed GBTM, Cox proportional-hazards models, floating absolute risk, and sensitivity analysis to conclude that solid cooking and heating fuel use was positively associated with the onset and exacerbation of FD. These statistical methods ensure the accuracy and comprehensiveness of our conclusions. Furthermore, we found that reducing solid household fuel can effectively prevent FD, which may provide valuable evidence for the reduction of the FD burden and the implementation of relevant policies.

## Limitations

Our study has several limitations. Firstly, we excluded a considerable proportion of participants at baseline due to missing data, which might have caused selection bias and affected the representativeness of our findings. Furthermore, indoor air pollution in this study was only assessed by the use of solid cooking and heating fuel, there might be other indoor pollutants that possess adverse health effects. Given that the ascertainments of fuel usage and FD were self-reported, potential misclassification might result in either an overestimation or an underestimation of the association between the two. For instance, the HR (95% CI) would be underestimated if participants with new-onset FD incorrectly reported being healthy. Some potential risk factors that may contribute to FD, such as cognitive function and trauma, were not included in this study owing to limited sample size or data constraints. Finally, due to data limitations, we only considered the fuel transition from 2011 to 2013, the subsequent transition between 2013 and 2018 was not assessed.

## Conclusion

In this cohort study, we found that solid cooking and heating fuel use was positively associated with the occurrence and exacerbation of FD. Switching from solid to clean household fuel, on the other hand, could significantly prevent FD, which emphasized the importance of universal access to clean energy advocated by the SDGs 7 and provided a viable direction for the development of healthy aging.

## Data availability statement

Publicly available datasets were analyzed in this study. This can be found here: http://charls.pku.edu.cn.

## Ethics statement

Ethical review was approved by the Institutional Review Board at Peking University. The patients/participants provided their written informed consent to participate in this study.

## Author contributions

PS and JL designed the study. ZR, LH, and SZ managed and analyzed the data. ZR and SS prepared the first draft. WS and ZR reviewed and edited the manuscript, with comments from PS, JL, CG, YW, and QY. PS had full access to the data and gave final approval of the submitted versions. All authors were involved in revising the paper, contributed to the article, and approved the submitted version.

## Conflict of interest

The authors declare that the research was conducted in the absence of any commercial or financial relationships that could be construed as a potential conflict of interest.

## Publisher's note

All claims expressed in this article are solely those of the authors and do not necessarily represent those of their affiliated organizations, or those of the publisher, the editors and the reviewers. Any product that may be evaluated in this article, or claim that may be made by its manufacturer, is not guaranteed or endorsed by the publisher.
