# Supplementary material for: Risk of functional disability associated with solid fuel use and population impact of reducing indoor air pollution in China: A national cohort study
Source: Front Public Health. 2022 Oct 3;10:976614. doi: 10.3389/fpubh.2022.976614 (PMC9575675; doi:10.3389/fpubh.2022.976614)
Supplement: Supplementary file 1 [file Data_Sheet_1.DOCX]

**Legends**

[Table S1. AIC and BIC of trajectory groups selection 2](#_Toc113447646)

[Table S2. Comparison of fuel usage among different ADL disability trajectory groups 3](#_Toc113447647)

[Table S3. Comparison of fuel usage among different IADL disability trajectory groups 4](#_Toc113447648)

[Table S4. Baseline characteristics of participants without ADL disability before 2011 in longitudinal analysis from 2011 to 2018 5](#_Toc113447649)

[Table S5. Baseline characteristics of participants without IADL disability before 2011 in longitudinal analysis from 2011 to 2018 7](#_Toc113447650)

[Table S6. Sensitivity analysis: HR (95% CI) of fuel usage with new-onset ADL and IADL disability in population without main chronic diseases 9](#_Toc113447651)

[Table S7. Sensitivity analysis: Logistic regression of fuel usage with new-onset ADL and IADL disability in total population 10](#_Toc113447652)

[Table S8. Baseline characteristics of participants without ADL disability before 2013 in longitudinal analysis from 2013 to 2018 11](#_Toc113447653)

[Table S9. Baseline characteristics of participants without IADL disability before 2013 in longitudinal analysis from 2013 to 2018 13](#_Toc113447654)

[Table S10. HR (95% CI) of fuel usage transitions with new-onset ADL disability: Cox proportional-hazards model 15](#_Toc113447655)

[Table S11. HR (95% CI) of fuel usage transitions with new-onset IADL disability: Cox proportional-hazards model 17](#_Toc113447656)

[Figure S1. ADL trajectory groups during 2011 and 2018 19](#_Toc113447657)

[Figure S2. IADL trajectory groups during 2011 and 2018 20](#_Toc113447658)

[Figure S3. The distribution of participants without ADL disability before 2011 in longitudinal analysis from 2011 to 2018 21](#_Toc113447659)

[Figure S4. The distribution of participants without IADL disability before 2011 in longitudinal analysis from 2011 to 2018 22](#_Toc113447660)

[Figure S5. Sankey diagram of household fuel usage transition from 2011 to 2013 and subsequent new-onset ADL disability 23](#_Toc113447661)

[Figure S6. Sankey diagram of household fuel usage transition from 2011 to 2013 and subsequent new-onset IADL disability 24](#_Toc113447662)

# Table S1. AIC and BIC of trajectory groups selection

|  | **Trajectory Groups** | | | |
| --- | --- | --- | --- | --- |
|  | **2** | **3** | **4** | **5** |
| **ADL** | | | | |
| AIC | -34042.83 | -33882.59 | -33861.94 | -33791.01 |
| BIC | -34071.06 | -33924.92 | -33918.39 | -33861.57 |
| **IADL** | | | | |
| AIC | -49582.03 | -49221.82 | -49150.51 | -49073.59 |
| BIC | -49612.65 | -49267.75 | -49211.75 | -49150.14 |

Notes: AIC, Akaike Information Criterion. BIC, Bayesian Information Criterion. ADL, activity of daily living. IADL, instrumental activity of daily living.

# Table S2. Comparison of fuel usage among different ADL disability trajectory groups

|  | **Maintained low ADL score** | **Increasing ADL score** | ***P* value** |
| --- | --- | --- | --- |
| **Cooking fuel** | | | <0.001 |
| Clean | 2317 (82.4) | 495 (17.6) |  |
| Solid | 4296 (74.6) | 1462 (25.4) |  |
| **Heating fuel** | | | <0.001 |
| Clean | 1088 (82.2) | 236 (17.8) |  |
| Solid | 5525 (76.3) | 1721 (23.8) |  |
| **Cooking and heating fuel** | | | <0.001 |
| Both clean | 859 (84.2) | 161 (15.8) |  |
| Cooking clean and heating solid | 1458 (81.4) | 334 (18.6) |  |
| Cooking solid and heating clean | 229 (75.3) | 75 (24.7) |  |
| Both solid | 4067 (74.6) | 1387 (25.4) |  |

Notes: ADL, activities of daily living. “Maintained low ADL score” group represents those maintaining low degree of ADL disability between 2011 and 2018. “Increasing ADL score” group represents those with exacerbating ADL disability between 2011 and 2018.

# Table S3. Comparison of fuel usage among different IADL disability trajectory groups

|  | **Maintained low IADL score** | **Increasing IADL score** | **P value** |
| --- | --- | --- | --- |
| **Cooking fuel** | | | <0.001 |
| Clean | 5337 (86.8) | 811 (13.2) |  |
| Solid | 7196 (76.2) | 2246 (23.8) |  |
| **Heating fuel** | | | <0.001 |
| Clean | 2751 (88.5) | 358 (11.5) |  |
| Solid | 9782 (78.4) | 2699 (21.6) |  |
| **Cooking and heating fuel** | | | <0.001 |
| Both clean | 2208 (89.3) | 264 (10.7) |  |
| Cooking clean and heating solid | 3129 (85.1) | 547 (14.9) |  |
| Cooking solid and heating clean | 543 (85.2) | 94 (14.8) |  |
| Both solid | 6653 (75.6) | 2152 (24.4) |  |

Notes: IADL, instrumental activities of daily living. “Maintained low IADL score” group represents those maintaining low degree of IADL disability between 2011 and 2018. “Increasing IADL score” group represents those with exacerbating IADL disability between 2011 and 2018.

# Table S4. Baseline characteristics of participants without ADL disability before 2011 in longitudinal analysis from 2011 to 2018

| **Baseline Characteristics** | | **New-onset ADL disability (*N*=2364)** | **No new-onset ADL disability (*N*=3852)** | ***P* value** |
| --- | --- | --- | --- | --- |
| Age, year | | 61.0 (55.0-68.0) | 58.0 (51.0-64.0) | <0.001 |
| Sex | |  |  | <0.001 |
|  | Male | 955 (40.4) | 1759 (45.7) |  |
|  | Female | 1409 (59.6) | 2093 (54.3) |  |
| Residence | | |  | 0.050 |
|  | Rural | 1676 (70.9) | 2640 (68.5) |  |
|  | Urban | 688 (29.1) | 1212 (31.5) |  |
| Education | | |  | <0.001 |
|  | Less than primary school | 1359 (57.5) | 1824 (47.4) |  |
|  | Primary school | 548 (23.2) | 965 (25.1) |  |
|  | Middle school | 327 (13.8) | 783 (20.3) |  |
|  | High school or above | 130 (5.5) | 280 (7.3) |  |
| Economic status | | |  |  |
|  | Bottom tertile | 855 (36.2) | 1328 (34.5) | 0.024 |
|  | Middle tertile | 845 (35.7) | 1316 (34.2) |  |
|  | Top tertile | 664 (28.1) | 1208 (31.4) |  |
| Marital status | |  |  | <0.001 |
|  | Married or cohabiting | 2038 (86.2) | 3488 (90.6) |  |
|  | Single | 326 (13.8) | 364 (9.5) |  |
| Cooking fuel | |  |  | <0.001 |
|  | Clean | 744 (31.5) | 1415 (36.7) |  |
|  | Solid | 1620 (68.5) | 2437 (63.3) |  |
| Heating fuel | |  |  | <0.001 |
|  | Clean | 339 (14.3) | 752 (19.5) |  |
|  | Solid | 2025 (85.7) | 3100 (80.5) |  |
| Cooking and heating fuel | |  |  | <0.001 |
|  | Both clean | 257 (10.9) | 579 (15.0) |  |
|  | Cooking clean and heating solid | 487 (20.6) | 836 (21.7) |  |
|  | Cooking solid and heating clean | 82 (3.5) | 173 (4.5) |  |
|  | Both solid | 1538 (65.1) | 2264 (58.8) |  |
| Smoking history | | |  | 0.108 |
|  | Never smoking | 1502 (63.5) | 2369 (61.5) |  |
|  | Ever smoking | 862 (36.5) | 1483 (38.5) |  |
| Drinking history | | |  | <0.001 |
|  | Never drinking | 1890 (80.0) | 2926 (76.0) |  |
|  | Ever drinking | 474 (20.1) | 926 (24.0) |  |
| BMI status | |  |  | 0.001 |
|  | Normal weight | 1362 (57.6) | 2275 (59.1) |  |
|  | Over weight | 664 (28.1) | 1148 (29.8) |  |
|  | Obesity | 338 (14.3) | 429 (11.1) |  |
| WC, cm | | 85.7 (78.0-93.0) | 84.4 (77.2-91.6) | <0.001 |
| Diabetes | |  |  |  |
|  | No | 1983 (83.9) | 3391 (88.0) | <0.001 |
|  | Yes | 381 (16.1) | 461 (12.0) |  |
| Hypertension | |  |  | <0.001 |
|  | No | 1270 (53.7) | 2315 (60.1) |  |
|  | Yes | 1094 (46.3) | 1537 (39.9) |  |
| Dyslipidemia | |  |  | 0.002 |
|  | No | 1706 (72.2) | 2919 (75.8) |  |
|  | Yes | 658 (27.8) | 933 (24.2) |  |
| CVDs | |  |  | <0.001 |
|  | No | 1931 (81.7) | 3362 (87.3) |  |
|  | Yes | 433 (18.3) | 490 (12.7) |  |
| Cognitive-related disorders | |  |  | 0.203 |
|  | No | 2336 (98.8) | 3819 (99.1) |  |
|  | Yes | 28 (1.2) | 33 (0.9) |  |

Notes: Values are presented as number (N) with percent (%) or medians with interquartile ranges (IQRs). ADL, activities of daily living. CVDs, cardiovascular diseases. BMI, body mass index. WC, waist circumference.

# Table S5. Baseline characteristics of participants without IADL disability before 2011 in longitudinal analysis from 2011 to 2018

| **Baseline Characteristics** | | **New-onset IADL disability (*N*=3309)** | **No new-onset IADL disability (*N*=6407)** | ***P* value** |
| --- | --- | --- | --- | --- |
| Age, year | | 60.0 (54.0-67.0) | 56.0 (49.0-62.0) | <0.001 |
| Sex | |  |  | <0.001 |
|  | Male | 1456 (44.0) | 3587 (56.0) |  |
|  | Female | 1853 (56.0) | 2820 (44.0) |  |
| Residence | | |  | <0.001 |
|  | Rural | 2391 (72.3) | 4050 (63.2) |  |
|  | Urban | 918 (27.7) | 2357 (36.8) |  |
| Education | | |  | <0.001 |
|  | Less than primary school | 1855 (56.1) | 2292 (35.8) |  |
|  | Primary school | 785 (23.7) | 1615 (25.2) |  |
|  | Middle school | 489 (14.8) | 1642 (25.6) |  |
|  | High school or above | 180 (5.4) | 858 (13.4) |  |
| Economic status | | |  | <0.001 |
|  | Bottom tertile | 1261 (38.1) | 1902 (29.7) |  |
|  | Middle tertile | 1151 (34.8) | 2179 (34.0) |  |
|  | Top tertile | 897 (27.1) | 2326 (36.3) |  |
| Marital status | |  |  | <0.001 |
|  | Married or cohabiting | 2943 (88.9) | 5934 (92.6) |  |
|  | Single | 366 (11.1) | 473 (7.4) |  |
| Cooking fuel | |  |  | <0.001 |
|  | Clean | 1035 (31.3) | 2854 (44.6) |  |
|  | Solid | 2274 (68.7) | 3553 (55.5) |  |
| Heating fuel | |  |  | <0.001 |
|  | Clean | 522 (15.8) | 1494 (23.3) |  |
|  | Solid | 2787 (84.2) | 4913 (76.7) |  |
| Cooking and heating fuel | |  |  | <0.001 |
|  | Both clean | 375 (11.3) | 1186 (18.5) |  |
|  | Cooking clean and heating solid | 660 (20.0) | 1668 (26.0) |  |
|  | Cooking solid and heating clean | 147 (4.4) | 308 (4.8) |  |
|  | Both solid | 2127 (64.3) | 3245 (50.7) |  |
| Smoking history | | |  | <0.001 |
|  | Never smoking | 2053 (62.0) | 3587 (56.0) |  |
|  | Ever smoking | 1256 (38.0) | 2820 (44.0) |  |
| Drinking history | | |  | <0.001 |
|  | Never drinking | 2568 (77.6) | 4539 (70.8) |  |
|  | Ever drinking | 741 (22.4) | 1868 (29.2) |  |
| BMI status | |  |  | 0.013 |
|  | Normal weight | 2006 (60.6) | 3768 (58.8) |  |
|  | Over weight | 892 (27.0) | 1958 (30.6) |  |
|  | Obesity | 411 (12.4) | 681 (10.6) |  |
| WC, cm | | 84.4 (77.3-91.6) | 84.3 (78.0-91.8) | 0.642 |
| Diabetes | |  |  | <0.001 |
|  | No | 2868 (86.7) | 5728 (89.4) |  |
|  | Yes | 441 (13.3) | 679 (10.6) |  |
| Hypertension | |  |  | <0.001 |
|  | No | 1898 (57.4) | 4151 (64.8) |  |
|  | Yes | 1411 (42.6) | 2256 (35.2) |  |
| Dyslipidemia | |  |  | <0.001 |
|  | No | 2455 (74.2) | 4983 (77.8) |  |
|  | Yes | 854 (25.8) | 1424 (22.2) |  |
| CVD | |  |  | <0.001 |
|  | No | 2817 (85.1) | 5883 (91.8) |  |
|  | Yes | 492 (14.9) | 524 (8.2) |  |
| Cognitive-related disorders | |  |  | <0.001 |
|  | No | 3277 (99.0) | 6373 (99.5) |  |
|  | Yes | 32 (1.0) | 34 (0.5) |  |

Notes: Values are presented as number (N) with percent (%) or medians with interquartile ranges (IQRs). IADL, instrumental activities of daily living. CVDs, cardiovascular diseases. BMI, body mass index.WC, waist circumference.

# Table S6. Sensitivity analysis: HR (95% CI) of fuel usage with new-onset ADL and IADL disability in population without main chronic diseases

|  | **ADL** | **IADL** |
| --- | --- | --- |
|  | **HR (95% CI)** | |
| Events/No. of participants | 800/2407 | 1285/4325 |
| ***Cooking fuel*** |  |  |
| Clean | Reference | Reference |
| Solid | 1.09 (0.93~1.28) | 1.26 (1.11~1.44) |
| ***Heating fuel*** |  |  |
| Clean | Reference | Reference |
| Solid | 1.16 (0.95~1.42) | 1.16 (0.99~1.35) |
| ***Cooking and heating fuel*** |  |  |
| Both clean | 1.00 (0.81~1.23) | 1.00 (0.84~1.18) |
| Cooking clean and heating solid | 1.09 (0.93~1.27) | 1.04 (0.92~1.18) |
| Cooking solid and heating clean | 0.93 (0.65~1.33) | 1.19 (0.93~1.51) |
| Both solid | 1.18 (1.07~1.31) | 1.33 (1.23~1.45) |

Notes: IADL, instrumental activities of daily living. HR, hazard ratio. CI, confidence interval. HR was adjusted for age, sex (male or female), residence (urban or rural), education (less than primary school, primary school, middle school, or high school or above), economic status (poor, middle, or rich), marital status (married or cohabiting, or single), smoking history (never smoking or ever smoking), drinking history (never drinking or ever drinking), body mass index status (normal weight, overweight, or obesity), waist circumference, diabetes (yes or no), hypertension (yes or no), dyslipidemia (yes or no), cardiovascular diseases (yes or no), and cognitive-related disorders (yes or no).

# Table S7. Sensitivity analysis: Logistic regression of fuel usage with new-onset ADL and IADL disability in total population

|  | **ADL** | **IADL** |
| --- | --- | --- |
|  | **OR (95% CI)** | |
| **No. of participants** | 1347/2420 | 2276/6076 |
| ***Cooking fuel*** | | |
| Clean | Reference | Reference |
| Solid | 1.24 (1.03~1.49) | 1.42 (1.26~1.60) |
| ***Heating fuel*** | | |
| Clean | Reference | Reference |
| Solid | 1.38 (1.09~1.76) | 1.18 (1.02~1.37) |
| ***Cooking and heating fuel*** | | |
| Both clean | Reference | Reference |
| Cooking clean and heating solid | 1.46 (1.08~1.97) | 0.99 (0.82~1.20) |
| Cooking solid and heating clean | 1.71 (0.99~2.98) | 1.24 (0.92~1.68) |
| Both solid | 1.59 (1.20~2.10) | 1.43 (1.19~1.70) |

Note: ADL, activities of daily living. IADL, instrumental activities of daily living. OR, odds ratio. CI, confidence interval. OR was adjusted for age, sex (male or female), residence (urban or rural), education (less than primary school, primary school, middle school, or high school or above), economic status (poor, middle, or rich), marital status (married or cohabiting, or single), smoking history (never smoking or ever smoking), drinking history (never drinking or ever drinking), body mass index status (normal weight, overweight, or obesity), waist circumference, diabetes (yes or no), hypertension (yes or no), dyslipidemia (yes or no), cardiovascular diseases (yes or no), and cognitive-related disorders (yes or no).

# Table S8. Baseline characteristics of participants without ADL disability before 2013 in longitudinal analysis from 2013 to 2018

| **Baseline Characteristics** | | **New-onset ADL disability (*N*=845)** | **No new-onset ADL disability (*N*=1800)** | ***P* value** |
| --- | --- | --- | --- | --- |
| Age, year | | 61.0 (55.0-68.0) | 59.0 (52.0-65.0) | <0.001 |
| Sex | |  |  | 0.004 |
|  | Male | 329 (38.9) | 807 (44.8) |  |
|  | Female | 516 (61.1) | 993 (55.2) |  |
| Residence | | |  | 0.165 |
|  | Rural | 616 (72.9) | 1265 (70.3) |  |
|  | Urban | 229 (27.1) | 535 (29.7) |  |
| Education | | |  | <0.001 |
|  | Less than primary school | 480 (56.8) | 859 (47.7) |  |
|  | Primary school | 193 (22.8) | 481 (26.7) |  |
|  | Middle school | 122 (14.4) | 351 (19.5) |  |
|  | High school or above | 50 (5.9) | 109 (6.1) |  |
| Economic status | | |  | 0.237 |
|  | Bottom tertile | 321 (38.0) | 670 (37.2) |  |
|  | Middle tertile | 308 (36.5) | 615 (34.2) |  |
|  | Top tertile | 216 (25.6) | 515 (28.6) |  |
| Marital status | |  |  | 0.001 |
|  | Married or cohabiting | 716 (84.7) | 1605 (89.2) |  |
|  | Single | 129 (15.3) | 195 (10.8) |  |
| Cooking fuel | |  |  | 0.002 |
|  | Clean | 241 (28.5) | 624 (34.7) |  |
|  | Solid | 604 (71.5) | 1176 (65.3) |  |
| Heating fuel | |  |  | <0.001 |
|  | Clean | 98 (11.6) | 324 (18.0) |  |
|  | Solid | 747 (88.4) | 1476 (82.0) |  |
| Cooking and heating fuel | |  |  | <0.001 |
|  | Both clean | 69 (8.2) | 251 (13.9) |  |
|  | Cooking clean and heating solid | 172 (20.4) | 373 (20.7) |  |
|  | Cooking solid and heating clean | 29 (3.4) | 73 (4.1) |  |
|  | Both solid | 575 (68.1) | 1103 (61.3) |  |
| Smoking history | | |  | 0.042 |
|  | Never smoking | 552 (65.3) | 1102 (61.2) |  |
|  | Ever smoking | 293 (34.7) | 698 (38.8) |  |
| Drinking history | | |  | 0.012 |
|  | Never drinking | 681 (80.6) | 1372 (76.2) |  |
|  | Ever drinking | 164 (19.4) | 428 (23.8) |  |
| BMI status | |  |  | 0.010 |
|  | Normal weight | 480 (56.8) | 1049 (58.3) |  |
|  | Over weight | 234 (27.7) | 546 (30.3) |  |
|  | Obesity | 131 (15.5) | 205 (11.4) |  |
| WC, cm | | 86.0 (78.0-93.4) | 84.8 (77.2-91.7) |  |
| Diabetes | |  |  | 0.013 |
|  | No | 715 (84.6) | 1586 (88.1) |  |
|  | Yes | 130 (15.4) | 214 (11.9) |  |
| Hypertension | |  |  | 0.059 |
|  | No | 468 (55.4) | 1067 (59.3) |  |
|  | Yes | 377 (44.6) | 733 (40.7) |  |
| Dyslipidemia | |  |  | 0.133 |
|  | No | 613 (72.5) | 1355 (75.3) |  |
|  | Yes | 232 (27.5) | 445 (24.7) |  |
| CVD | |  |  | <0.001 |
|  | No | 679 (80.4) | 1555 (86.4) |  |
|  | Yes | 166 (19.6) | 245 (13.6) |  |
| Cognitive-related disorders | |  |  | 0.213 |
|  | No | 833 (98.6) | 1784 (99.1) |  |
|  | Yes | 12 (1.4) | 16 (0.9) |  |

Notes: Values are presented as number (N) with percent (%) or medians with interquartile ranges (IQRs). ADL, activities of daily living. CVDs, cardiovascular diseases. BMI, body mass index. WC, waist circumference.

# Table S9. Baseline characteristics of participants without IADL disability before 2013 in longitudinal analysis from 2013 to 2018

| **Baseline Characteristics** | | **New-onset IADL disability (N=1351)** | **No new-onset IADL disability (N=4202)** | ***P* value** |
| --- | --- | --- | --- | --- |
| Age, year | | 59.0 (53.0-66.0) | 56.0 (49.0-62.0) | <0.001 |
| Sex | |  |  | <0.001 |
|  | Male | 585 (43.3) | 2415 (57.5) |  |
|  | Female | 766 (56.7) | 1787 (42.5) |  |
| Residence | | |  | <0.001 |
|  | Rural | 1000 (74.0) | 2782 (66.2) |  |
|  | Urban | 351 (26.0) | 1420 (33.8) |  |
| Education | | |  | <0.001 |
|  | Less than primary school | 713 (52.8) | 1466 (34.9) |  |
|  | Primary school | 344 (25.5) | 1074 (25.6) |  |
|  | Middle school | 220 (16.3) | 1099 (26.2) |  |
|  | High school or above | 74 (5.5) | 563 (13.4) |  |
| Economic status | | |  | <0.001 |
|  | Bottom tertile | 516 (38.2) | 1312 (31.2) |  |
|  | Middle tertile | 473 (35.0) | 1382 (32.9) |  |
|  | Top tertile | 362 (26.8) | 1508 (35.9) |  |
| Marital status | |  |  | <0.001 |
|  | Married or cohabiting | 1189 (88.0) | 3864 (92.0) |  |
|  | Single | 162 (12.0) | 338 (8.0) |  |
| Cooking fuel | |  |  | <0.001 |
|  | Clean | 423 (31.3) | 1810 (43.1) |  |
|  | Solid | 928 (68.7) | 2392 (56.9) |  |
| Heating fuel | |  |  | <0.001 |
|  | Clean | 223 (16.5) | 930 (22.1) |  |
|  | Solid | 1128 (83.5) | 3272 (77.9) |  |
| Cooking and heating fuel | |  |  | <0.001 |
|  | Both clean | 156 (11.6) | 736 (17.5) |  |
|  | Cooking clean and heating solid | 267 (19.8) | 1074 (25.6) |  |
|  | Cooking solid and heating clean | 67 (5.0) | 194 (4.6) |  |
|  | Both solid | 861 (63.7) | 2198 (52.3) |  |
| Smoking history | | |  | <0.001 |
|  | No smoking | 836 (61.9) | 2352 (56.0) |  |
|  | Smoking | 515 (38.1) | 1850 (44.0) |  |
| Drinking history | | |  | <0.001 |
|  | No drinking | 1047 (77.5) | 2953 (70.3) |  |
|  | Drinking | 304 (22.5) | 1249 (29.7) |  |
| BMI status | |  |  | 0.017 |
|  | Normal weight | 789 (58.4) | 2489 (59.2) |  |
|  | Over weight | 387 (28.7) | 1282 (30.5) |  |
|  | Obesity | 175 (13.0) | 431 (10.3) |  |
| WC, cm | | 84.6 (77.3-92.0) | 84.0 (77.8-91.0) | 0.341 |
| Diabetes | |  |  | <0.001 |
|  | No | 1162 (86.0) | 3754 (89.3) |  |
|  | Yes | 189 (14.0) | 448 (10.7) |  |
| Hypertension | |  |  | <0.001 |
|  | No | 764 (56.6) | 2784 (66.3) |  |
|  | Yes | 587 (43.5) | 1418 (33.8) |  |
| Dyslipidemia | |  |  | <0.001 |
|  | No | 965 (71.4) | 3277 (78.0) |  |
|  | Yes | 386 (28.6) | 925 (22.0) |  |
| CVD | |  |  | <0.001 |
|  | No | 1165 (86.2) | 3881 (92.4) |  |
|  | Yes | 186 (13.8) | 321 (7.6) |  |
| Cognitive-related disorders | |  |  | 0.913 |
|  | No | 1343 (99.4) | 4176 (99.4) |  |
|  | Yes | 8 (0.6) | 26 (0.6) |  |

Notes: Values are presented as number (N) with percent (%) or medians with interquartile ranges (IQRs). IADL, instrumental activities of daily living. CVD, cardiovascular disease. BMI, body mass index. WC, waist circumference.

# Table S10. HR (95% CI) of fuel usage transitions with new-onset ADL disability: Cox proportional-hazards model

|  | **Events/No. of participants** | **Total population (N=2645)** | **Age-stratified** | | **Sex-stratified** | | **Residence-stratified** | |
| --- | --- | --- | --- | --- | --- | --- | --- | --- |
|  |  |  | **Age < 65 (N=1868)** | **Age ≥ 65 (N=777)** | **Male (N=1136)** | **Female (N=1509)** | **Rural (N=1881)** | **Urban (N=764)** |
| ***Cooking fuel*** |  |  |  |  |  |  |  |  |
| Keep clean | 189/687 | 1.00 (0.85~1.17) | 1.00 (0.83~1.20) | 1.00 (0.73~1.37) | 1.00 (0.77~1.30) | 1.00 (0.82~1.22) | 1.00 (0.80~1.25) | 1.00 (0.82~1.22) |
| Clean to solid | 52/178 | 1.06 (0.81~1.40) | 0.95 (0.67~1.35) | 1.33 (0.87~2.05) | 0.98 (0.62~1.54) | 1.02 (0.72~1.44) | 1.24 (0.90~1.70) | 0.78 (0.45~1.35) |
| Keep solid | 458/1316 | 1.00 (0.91~1.10) | 1.00 (0.88~1.13) | 1.00 (0.85~1.18) | 1.00 (0.85~1.17) | 1.00 (0.88~1.14) | 1.00 (0.90~1.11) | 1.00 (0.79~1.26) |
| Solid to clean | 146/464 | 0.88 (0.75~1.03) | 0.91 (0.74~1.11) | 0.85 (0.65~1.12) | 0.90 (0.69~1.17) | 0.90 (0.73~1.10) | 0.94 (0.79~1.13) | 0.64 (0.44~0.93) |
| ***Heating fuel*** |  |  |  |  |  |  |  |  |
| Keep clean | 43/220 | 1.00 (0.73~1.36) | 1.00 (0.65~1.53) | 1.00 (0.63~1.59) | 1.00 (0.56~1.8) | 1.00 (0.69~1.44) | 1.00 (0.57~1.77) | 1.00 (0.70~1.43) |
| Clean to solid | 55/202 | 1.49 (1.14~1.94) | 2.18 (1.60~2.97) | 0.82 (0.49~1.38) | 2.09 (1.34~3.25) | 1.24 (0.89~1.73) | 1.54 (1.10~2.16) | 1.33 (0.86~2.05) |
| Keep solid | 652/1901 | 1.00 (0.91~1.09) | 1.00 (0.90~1.11) | 1.00 (0.85~1.18) | 1.00 (0.87~1.15) | 1.00 (0.89~1.13) | 1.00 (0.91~1.10) | 1.00 (0.84~1.20) |
| Solid to clean | 95/322 | 0.80 (0.66~0.98) | 0.86 (0.67~1.10) | 0.71 (0.51~0.99) | 1.00 (0.75~1.35) | 0.68 (0.52~0.89) | 0.77 (0.60~0.98) | 0.88 (0.63~1.25) |
| ***Cooking and heating fuel*** | |  |  |  |  |  |  |  |
| Keep both clean | 34/174 | 1.00 (0.71~1.42) | 1.00 (0.63~1.58) | 1.00 (0.57~1.75) | 1.00 (0.54~1.85) | 1.00 (0.65~1.53) | 1.00 (0.47~2.11) | 1.00 (0.68~1.48) |
| Both clean to 1 solid | 27/126 | 1.21 (0.83~1.77) | 1.63 (1.05~2.53) | 0.76 (0.36~1.62) | 1.83 (1.00~3.33) | 1.01 (0.62~1.66) | 1.13 (0.62~2.05) | 1.32 (0.80~2.16) |
| Both clean to both solid | 8/20 | 1.98 (0.98~3.98) | 3.53 (1.31~9.53) | 1.22 (0.45~3.31) | 2.97 (1.19~7.37) | 1.05 (0.34~3.29) | 2.28 (1.01~5.17) | 1.43 (0.35~5.83) |
| Keep 1 solid | 111/359 | 1.00 (0.83~1.21) | 1.00 (0.80~1.24) | 1.00 (0.67~1.50) | 1.00 (0.71~1.40) | 1.00 (0.79~1.26) | 1.00 (0.77~1.30) | 1.00 (0.76~1.32) |
| 1 solid to both clean | 35/117 | 0.90 (0.64~1.25) | 0.84 (0.56~1.27) | 0.98 (0.53~1.79) | 1.35 (0.82~2.24) | 0.68 (0.43~1.07) | 0.80 (0.47~1.35) | 0.95 (0.61~1.48) |
| 1 solid to both solid | 55/171 | 1.00 (0.77~1.31) | 0.88 (0.63~1.22) | 1.29 (0.82~2.03) | 0.86 (0.53~1.40) | 1.01 (0.73~1.39) | 1.12 (0.83~1.52) | 0.75 (0.43~1.30) |
| Keep both solid | 417/1176 | 1.00 (0.90~1.11) | 1.00 (0.88~1.14) | 1.00 (0.84~1.19) | 1.00 (0.84~1.18) | 1.00 (0.88~1.14) | 1.00 (0.90~1.11) | 1.00 (0.78~1.28) |
| Both solid to both clean | 39/124 | 0.81 (0.59~1.11) | 0.87 (0.58~1.30) | 0.77 (0.46~1.28) | 1.04 (0.67~1.63) | 0.67 (0.43~1.05) | 0.85 (0.59~1.22) | 0.73 (0.38~1.42) |
| Both solid to 1 solid | 119/378 | 0.86 (0.71~1.03) | 0.91 (0.73~1.14) | 0.77 (0.56~1.05) | 0.74 (0.54~1.02) | 0.95 (0.76~1.18) | 0.89 (0.73~1.09) | 0.65 (0.42~1.00) |

Notes: ADL, activities of daily living. HR, hazard ratio. CI, confidence interval. 1 solid, either solid cooking fuel or solid heating fuel. HR was adjusted for age (continuous value), sex (male or female), residence (urban or rural), education (less than primary school, primary school, middle school, or high school or above), economic status (poor, middle, or rich), marital status (married or cohabiting, or single), smoking history (never smoking or ever smoking), drinking history (never drinking or ever drinking), body mass index status (normal weight, overweight, or obesity), waist circumference, diabetes (yes or no), hypertension (yes or no), dyslipidemia (yes or no), cardiovascular diseases (yes or no), and cognitive-related disorders (yes or no). The reference of "Clean to solid" is "Keep clean"; the reference of "Solid to clean" is "Keep solid"; the reference of "Both clean to 1 solid" and "Both clean to both solid" is "Keep both clean"; the reference of "1 solid to both clean" and "1 solid to both solid" is "Keep 1 solid"; the reference of "Both solid to both clean" and "Both solid to 1 solid" is "Keep both solid".

# Table S11. HR (95% CI) of fuel usage transitions with new-onset IADL disability: Cox proportional-hazards model

|  | **Cases/No. of participants** | **Total population (N=5553)** | **Age-stratified** | | **Sex-stratified** | | **Residence-stratified** | |
| --- | --- | --- | --- | --- | --- | --- | --- | --- |
|  |  |  | **Age < 65 (N=4398)** | **Age≥ 65 (N=1155)** | **Male (N=3000)** | **Female (N=2553)** | **Rural (N=3782)** | **Urban (N=1771)** |
| ***Cooking fuel*** |  |  |  |  |  |  |  |  |
| Keep clean | 326/1848 | 1.00 (0.89~1.13) | 1.00 (0.87~1.14) | 1.00 (0.78~1.28) | 1.00 (0.83~1.20) | 1.00 (0.85~1.17) | 1.00 (0.86~1.17) | 1.00 (0.84~1.18) |
| Clean to solid | 97/385 | 1.29 (1.05~1.57) | 1.13 (0.89~1.43) | 1.92 (1.33~2.78) | 1.56 (1.16~2.10) | 1.11 (0.85~1.46) | 1.28 (1.01~1.62) | 1.27 (0.86~1.88) |
| Keep solid | 699/2378 | 1.00 (0.92~1.08) | 1.00 (0.91~1.10) | 1.00 (0.86~1.16) | 1.00 (0.88~1.13) | 1.00 (0.90~1.11) | 1.00 (0.92~1.09) | 1.00 (0.83~1.20) |
| Solid to clean | 229/942 | 0.84 (0.74~0.96) | 0.88 (0.76~1.02) | 0.76 (0.59~0.99) | 0.89 (0.73~1.09) | 0.81 (0.68~0.95) | 0.90 (0.78~1.04) | 0.66 (0.50~0.87) |
| ***Heating fuel*** |  |  |  |  |  |  |  |  |
| Keep clean | 91/613 | 1.00 (0.81~1.23) | 1.00 (0.77~1.30) | 1.00 (0.69~1.45) | 1.00 (0.74~1.36) | 1.00 (0.75~1.34) | 1.00 (0.64~1.57) | 1.00 (0.79~1.27) |
| Clean to solid | 132/540 | 1.59 (1.34~1.89) | 1.79 (1.47~2.17) | 1.19 (0.81~1.73) | 1.14 (0.86~1.52) | 2.04 (1.64~2.53) | 3.48 (2.82~4.29) | 0.94 (0.69~1.27) |
| Keep solid | 966/3646 | 1.00 (0.93~1.08) | 1.00 (0.92~1.09) | 1.00 (0.86~1.16) | 1.00 (0.89~1.12) | 1.00 (0.91~1.10) | 1.00 (0.93~1.08) | 1.00 (0.86~1.16) |
| Solid to clean | 162/754 | 0.83 (0.71~0.96) | 0.89 (0.75~1.07) | 0.64 (0.47~0.86) | 0.77 (0.61~0.98) | 0.87 (0.71~1.06) | 0.84 (0.70~1.02) | 0.79 (0.61~1.04) |
| ***Cooking and heating fuel*** | |  |  |  |  |  |  |  |
| Keep both clean | 69/475 | 1.00 (0.78~1.28) | 1.00 (0.75~1.33) | 1.00 (0.62~1.61) | 1.00 (0.69~1.44) | 1.00 (0.72~1.39) | 1.00 (0.59~1.69) | 1.00 (0.76~1.32) |
| Both clean to 1 solid | 73/352 | 1.37 (1.09~1.74) | 1.41 (1.08~1.84) | 1.33 (0.80~2.19) | 1.28 (0.89~1.82) | 1.47 (1.07~2.00) | 2.58 (1.87~3.56) | 1.02 (0.73~1.42) |
| Both clean to both solid | 14/65 | 1.27 (0.75~2.15) | 1.10 (0.57~2.11) | 2.29 (0.94~5.58) | 1.25 (0.52~3.03) | 1.32 (0.68~2.55) | 2.94 (1.66~5.19) | 0.40 (0.10~1.63) |
| Keep 1 solid | 157/823 | 1.00 (0.85~1.17) | 1.00 (0.83~1.20) | 1.00 (0.72~1.38) | 1.00 (0.78~1.29) | 1.00 (0.82~1.23) | 1.00 (0.82~1.22) | 1.00 (0.77~1.30) |
| 1 solid to both clean | 72/412 | 0.90 (0.72~1.14) | 1.02 (0.78~1.33) | 0.63 (0.39~1.02) | 0.90 (0.63~1.28) | 0.91 (0.67~1.24) | 0.95 (0.71~1.29) | 0.87 (0.60~1.25) |
| 1 solid to both solid | 105/367 | 1.43 (1.18~1.74) | 1.42 (1.14~1.78) | 1.40 (0.96~2.04) | 1.49 (1.11~2.01) | 1.38 (1.07~1.78) | 1.33 (1.06~1.65) | 1.71 (1.15~2.53) |
| Keep both solid | 617/2083 | 1.00 (0.92~1.09) | 1.00 (0.90~1.11) | 1.00 (0.85~1.18) | 1.00 (0.88~1.14) | 1.00 (0.89~1.12) | 1.00 (0.92~1.09) | 1.00 (0.81~1.24) |
| Both solid to both clean | 52/232 | 0.76 (0.58~0.99) | 0.80 (0.58~1.12) | 0.69 (0.42~1.13) | 0.79 (0.52~1.20) | 0.73 (0.51~1.05) | 0.74 (0.53~1.03) | 0.74 (0.46~1.19) |
| Both solid to 1 solid | 192/744 | 0.87 (0.75~1.00) | 0.91 (0.77~1.06) | 0.72 (0.53~0.97) | 0.94 (0.76~1.17) | 0.83 (0.68~0.99) | 0.91 (0.78~1.06) | 0.69 (0.50~0.95) |

Notes: IADL, instrumental activities of daily living. HR, hazard ratio. CI, confidence interval. 1 solid, either solid cooking fuel or solid heating fuel. HR was adjusted for age (continuous value), sex (male or female), residence (urban or rural), education (less than primary school, primary school, middle school, or high school or above), economic status (poor, middle, or rich), marital status (married or cohabiting, or single), smoking history (never smoking or ever smoking), drinking history (never drinking or ever drinking), body mass index status (normal weight, overweight, or obesity), waist circumference, diabetes (yes or no), hypertension (yes or no), dyslipidemia (yes or no), cardiovascular diseases (yes or no), and cognitive-related disorders (yes or no). The reference of "Clean to solid" is "Keep clean"; the reference of "Solid to clean" is "Keep solid"; the reference of "Both clean to 1 solid" and "Both clean to both solid" is "Keep both clean"; the reference of "1 solid to both clean" and "1 solid to both solid" is "Keep 1 solid"; the reference of "Both solid to both clean" and "Both solid to 1 solid" is "Keep both solid".


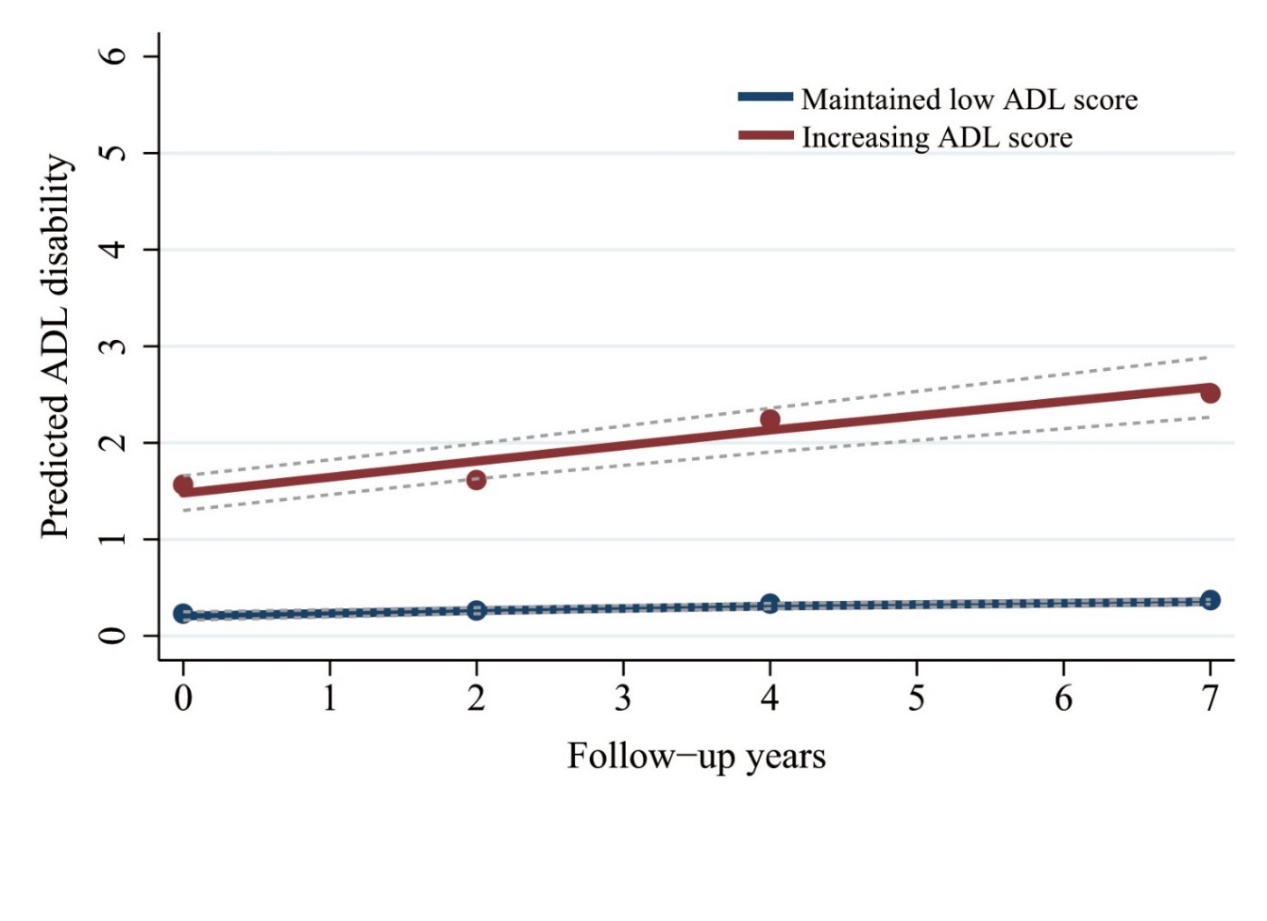


# Figure S1. ADL trajectory groups during 2011 and 2018

Notes: ADL, activity of daily living. “Maintained low ADL score” group represents those maintaining low degree of ADL disability between 2011 and 2018. “Increasing ADL score” group represents those with exacerbating ADL disability between 2011 and 2018.


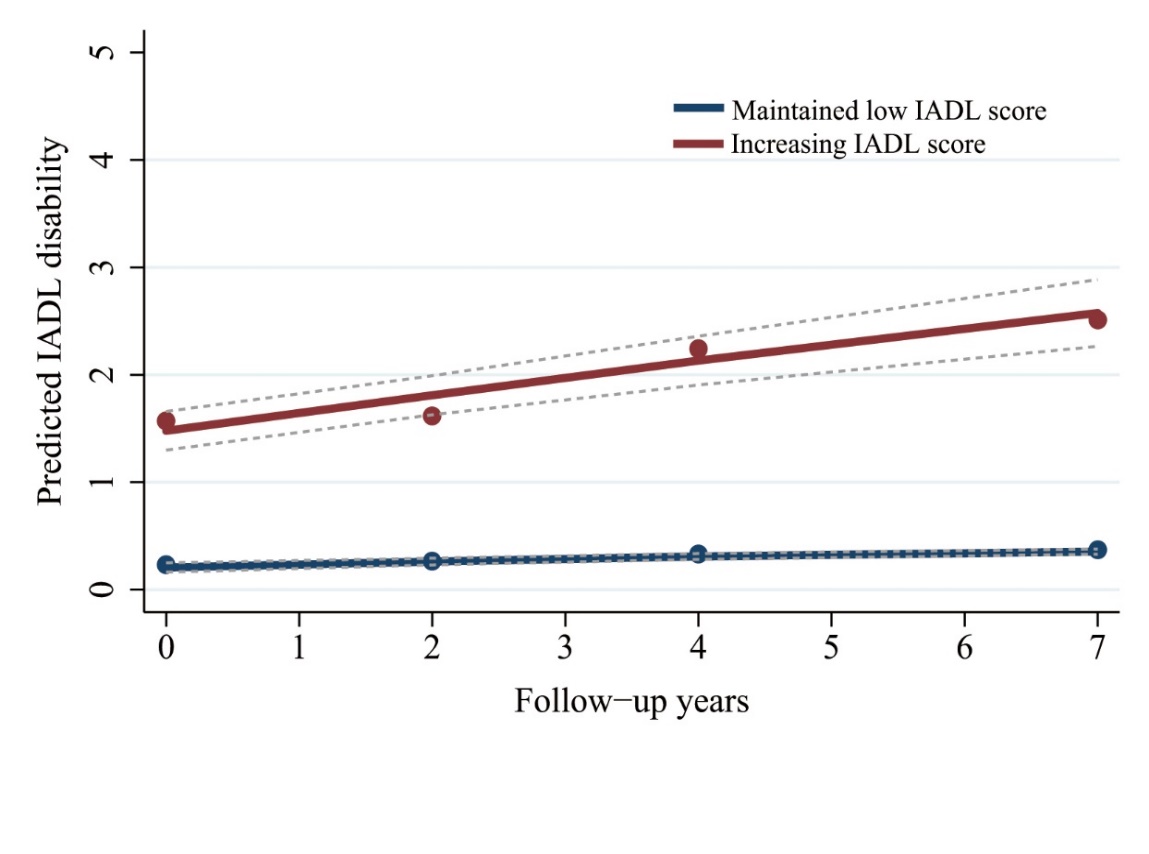


# Figure S2. IADL trajectory groups during 2011 and 2018

Notes: IADL, instrumental activity of daily living. “Maintained low IADL score” group represents those maintaining low degree of IADL disability between 2011 and 2018. “Increasing IADL score” group represents those with exacerbating IADL disability between 2011 and 2018.


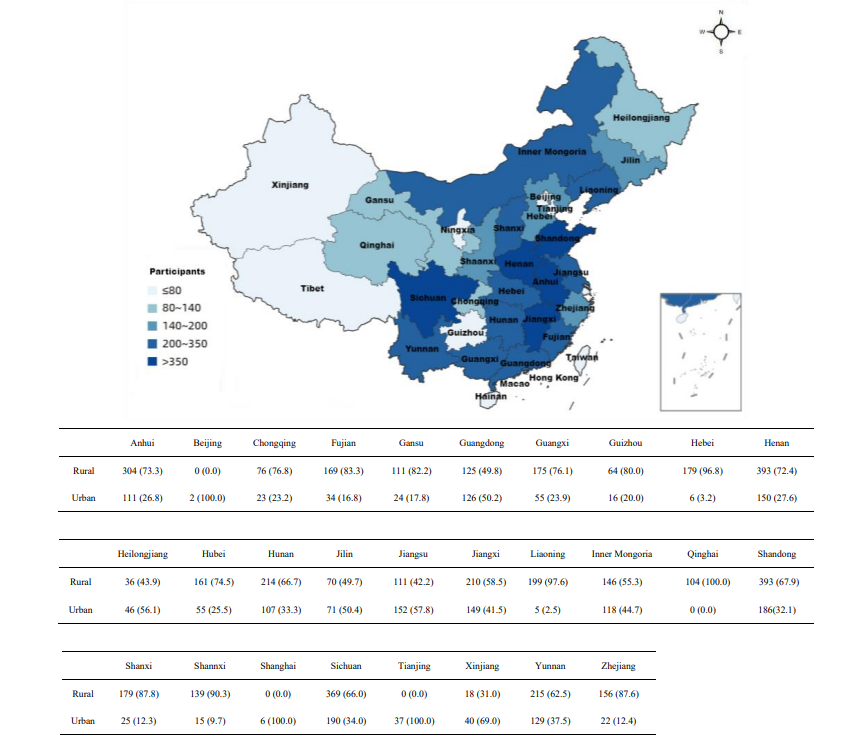


# Figure S3. The distribution of participants without ADL disability before 2011 in longitudinal analysis from 2011 to 2018


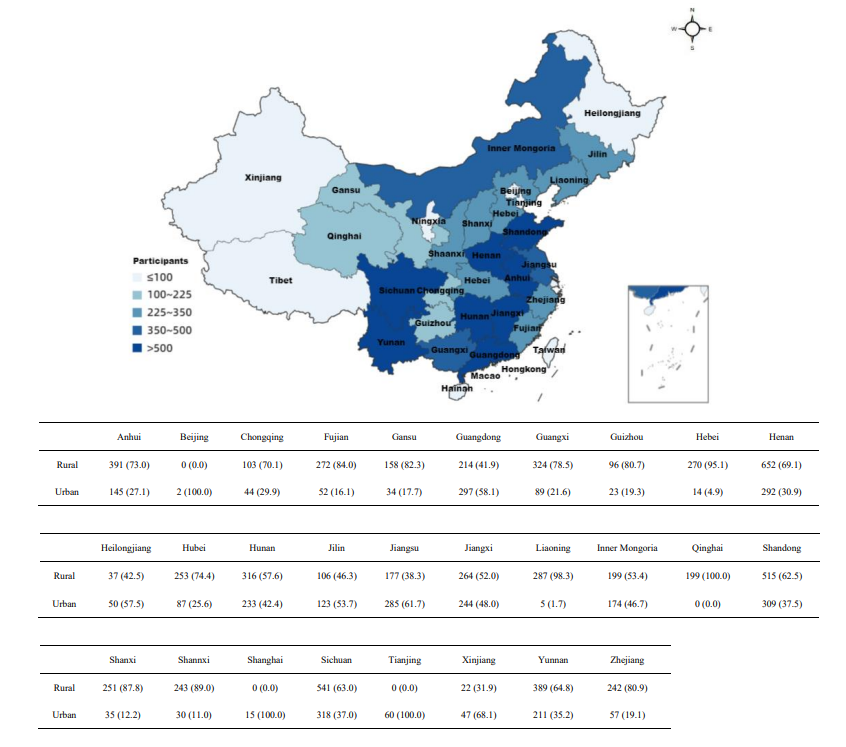


# Figure S4. The distribution of participants without IADL disability before 2011 in longitudinal analysis from 2011 to 2018


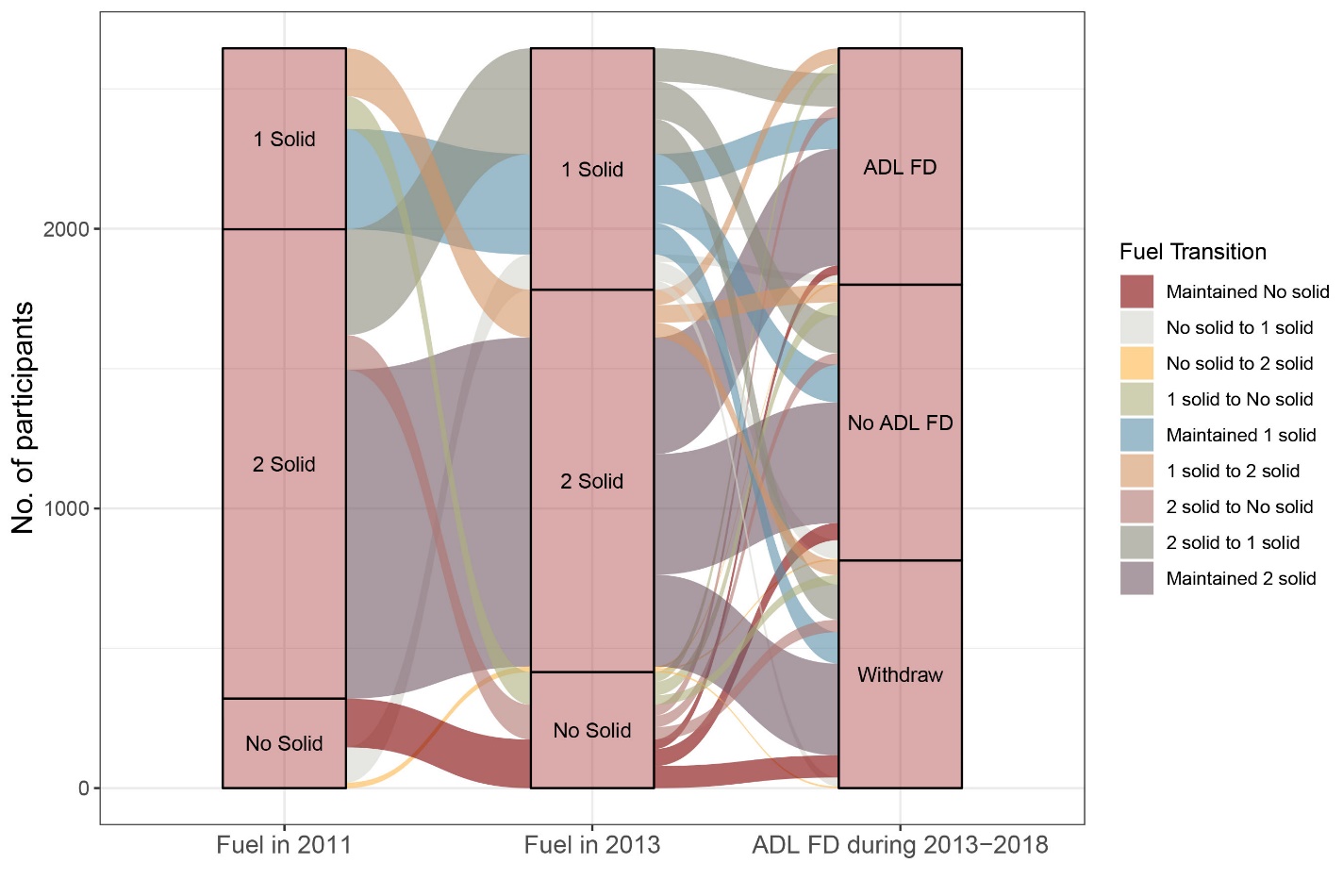


# Figure S5. Sankey diagram of household fuel usage transition from 2011 to 2013 and subsequent new-onset ADL disability

Notes: ADL, activity of daily living. FD, functional disability. 1 solid, either solid cooking fuel or solid heating fuel. 2 solid, both solid cooking fuel and solid heating fuel. No solid, both clean cooking fuel and clean heating fuel.


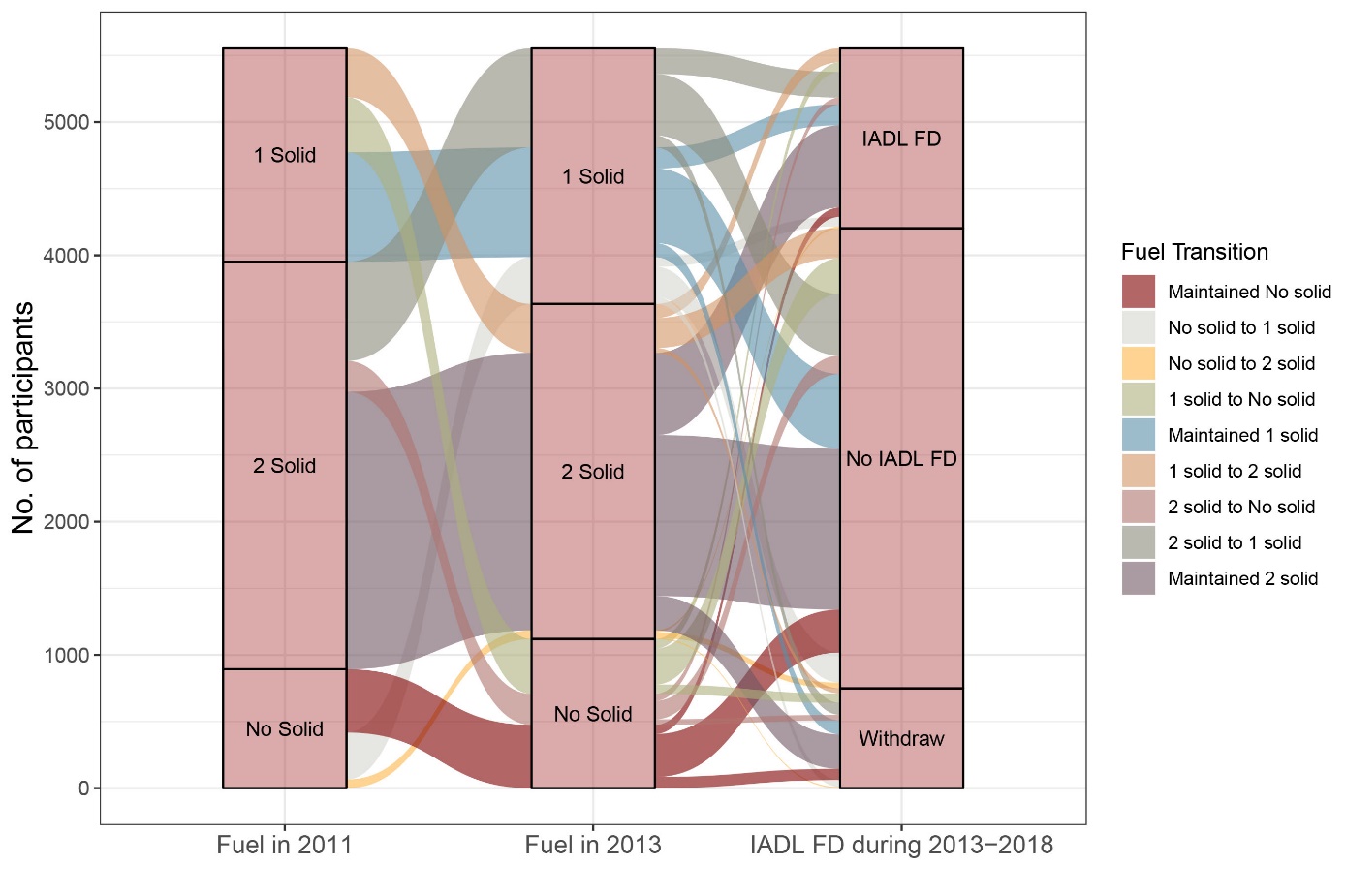


# Figure S6. Sankey diagram of household fuel usage transition from 2011 to 2013 and subsequent new-onset IADL disability

Notes: IADL, instrumental activity of daily living. FD, functional disability. 1 solid, either solid cooking fuel or solid heating fuel. 2 solid, both solid cooking fuel and solid heating fuel. No solid, both clean cooking fuel and clean heating fuel.
